# Clinical and demographic characteristics of COVID-19 cases in Brunei Darussalam: comparison between the first and second waves, 2020 and 2021

**DOI:** 10.5365/wpsar.2022.13.3.925

**Published:** 2022-08-25

**Authors:** Muhammad Umer Malik, Muhammad Syafiq Abdullah, Pui Lin Chong, Rosmonaliza Asli, Babu Ivan Mani, Nooraffizan Rahman, Natalie Riamiza Momin, Chin Ann Lim, Justin Wong, Chee Fui Chong, Vui Heng Chong

**Affiliations:** a National Isolation Centre, Tutong, Brunei Darussalam.; b Pengiran Anak Puteri Rashidah Sa’adatul Bolkiah Institute of Health Sciences, Universiti Brunei Darussalam, Bandar Seri Begawan, Brunei Darussalam.; c Department of Public Health, Ministry of Health, Bandar Seri Begawan, Brunei Darussalam.

## Abstract

**Objective:**

Differences in clinical manifestations between strains of severe acute respiratory syndrome coronavirus 2 (SARS-CoV-2) have been reported. This retrospective descriptive study compares the clinical and demographic characteristics of all confirmed coronavirus disease (COVID-19) cases admitted to the National Isolation Centre (NIC) in the first wave and at the beginning of the second wave of the pandemic in Brunei Darussalam.

**Methods:**

All COVID-19 cases admitted to the NIC between 9 March and 6 May 2020 (first wave) and 7–17 August 2021 (second wave) were included. Data were obtained from NIC databases and case characteristics compared using Student’s *t*-tests and χ^2^ tests, as appropriate.

**Results:**

Cases from the first wave were significantly older than those from the second wave (mean 37.2 vs 29.7 years, *P* < 0.001), and a higher proportion reported comorbidities (30.5% vs 20.3%, *P* = 0.019). Cases from the second wave were more likely to be symptomatic at admission (77.7% vs 63.1%, *P* < 0.001), with a higher proportion reporting cough, anosmia, sore throat and ageusia/dysgeusia; however, myalgia and nausea/vomiting were more common among symptomatic first wave cases (all *P* < 0.05). There was no difference in the mean number of reported symptoms (2.6 vs 2.4, *P* = 0.890).

**Discussion:**

Our study showed clear differences in the profile of COVID-19 cases in Brunei Darussalam between the first and second waves, reflecting a shift in the predominating SARS-CoV-2 strain. Awareness of changes in COVID-19 disease manifestation can help guide adjustments to management policies such as duration of isolation, testing strategies, and criteria for admission and treatment.

The emergence and rapid spread of severe acute respiratory syndrome coronavirus 2 (SARS-CoV-2), the causal pathogen of the coronavirus disease (COVID-19) pandemic, has presented health services with major challenges and has disrupted social and economic activities worldwide. As of 15 August 2022, the World Health Organization (WHO) had recorded over 587 million confirmed cases and over 6.4 million deaths due to COVID-19 worldwide. (*1*) In Brunei Darussalam, the first wave of the COVID-19 pandemic started on 9 March 2020 and lasted until 6 May 2020, the date of the last documented case of community spread. After approximately 15 months of being at WHO Level 2 transmission (low community incidence or a risk of community transmission beyond clusters), (*2*) a second wave, confirmed to be due to the Delta strain (SARS-CoV-2 variant B.1.617.2), started on 7 August 2021.

Compared with the original SARS-CoV-2 strain, the Delta variant has a higher reproduction number (R^0^) and was the dominant variant circulating in Brunei Darussalam during 2021 when this study was conducted. (*3*) Differences in the clinical characteristics of cases due to the original and Delta strains of the virus have been widely reported in the literature, although these studies have been restricted to patients needing hospital admission. (*4*-*8*) To date, few studies have compared the first and second waves across the full spectrum of COVID-19 disease severity, i.e. by including asymptomatic and symptomatic patients as well as those with more severe disease. Understanding differences between the two waves in disease presentation can help improve the management of patients.

During the first wave in Brunei Darussalam, all confirmed COVID-19 patients were admitted to the National Isolation Centre (NIC) for isolation and treatment. During the second wave, community isolation centres (CICs) with minimal medical facilities were used to care for asymptomatic or mild cases (i.e. symptomatic cases that did not need specific treatment). Recovering patients admitted to the NIC were also transferred to CICs until they fulfilled criteria for discharge, and patients admitted to CICs whose condition subsequently deteriorated were transferred to the NIC. Until the CICs were opened on 18 August 2021, the NIC remained the only designated isolation and treatment centre in Brunei Darussalam for all confirmed cases of COVID-19.

The objective of this study was to compare the differences between the first and second waves in the clinical and demographic characteristics of all confirmed COVID-19 cases in Brunei Darussalam, including asymptomatic, mild and severe cases.

## Methods

### Study participants

All cases admitted to the NIC between 9 March and 6 May 2020 (the first wave) and between 7 and 17 August 2021 (the second wave) were included in the study. Subjects admitted to the NIC after 18 August were not included in this study, as from this date onwards asymptomatic and mild cases of COVID-19 were instead admitted to CICs. Inclusion of subjects admitted to the NIC after 18 August would have led to a second wave study population that was biased towards more severe cases and thus not representative of the complete spectrum of COVID-19 disease severity.

### Data collection

Case finding and contact tracing were conducted by the Department of Public Health, and all confirmed COVID-19 cases were registered and assigned a unique case identification number. Data for all cases were retrieved from prospectively maintained Excel databases, created by the various teams set up by the Ministry of Health’s National COVID-19 Committee to help with the management of patients. Data collected included information on patient demographics, comorbidities, reported COVID-19 symptoms, disease progression and outcomes. Each patient was asked to complete a detailed symptom checklist on admission to the NIC, which included questions about symptom onset.

### Case definitions

#### Symptom category

Cases were categorized as (i) asymptomatic (no symptoms experienced during course of illness), (ii) pre-symptomatic (no symptoms at NIC admission but developed symptoms later), (iii) symptomatic (symptoms at NIC admission) and (iv) recovered (symptoms resolved before NIC admission). Patients were assigned to a symptom category according to their status at the time of their admission to the NIC.

#### Disease category

For the purposes of this study, four categories of disease were defined: (i) asymptomatic/mild (no symptoms or symptomatic without evidence of pneumonia on chest imaging), (ii) moderate (clinical or imaging evidence of pneumonia), (iii) severe (required oxygen supplementation) and (iv) critical (respiratory failure requiring mechanical ventilation with or without other organ failure). Patients’ disease categories were assessed daily and reported to the Ministry of Health. Patients were assigned to the highest category reached during the course of their illness.

### Data analysis

Patient data were anonymized before analysis. Clinical and demographic characteristics of patients from the two waves were compared; tests for statistical significance of differences between the two cohorts were performed as appropriate (Student’s *t*-test for continuous variables and χ^2^ test for categorical variables). *P*-values of < 0.05 were considered statistically significant. Analyses were conducted using SPSS version 26.0.

## Results

During the first wave of the COVID-19 pandemic, a total of 141 cases were admitted to the NIC. During 7–17 August 2021, the period of the second wave included in this study, 359 cases were admitted. COVID-19 cases from the first wave were significantly older and were more likely to have cardiovascular comorbidities compared with those in the second wave (**Table 1**).

**Table 1:** Demographic and clinical characteristics of COVID-19 cases in the first and second wave,  Brunei Darussalam, 2020–2021

Characteristic	First wave (*n* = 141)	Second wave (*n* = 359)	*P*
Age (years; mean ± SD)	37.2 ± 17.4	29.7 ± 16.6	< 0.001
Age group (years)			
< 13	12 (8.5)	47 (13.1)	< 0.001
13–18	9 (6.4)	70 (19.5)	-
19–29	28 (19.9)	76 (21.2)	-
30–39	30 (21.3)	57 (15.9)	-
40–49	20 (14.2)	62 (17.3)	-
50–59	25 (17.7)	29 (8.1)	-
^3^60	17 (12.1)	18 (5.0)	-
Sex			
Male	85 (60.3)	190 (52.9)	0.137
Female	56 (39.7)	169 (47.1)	-
Pregnant patients	2 (1.4)	8 (2.2)	0.560
Comorbidity (at least one)	43 (30.5)	73 (20.3)	0.015
Diabetes mellitus	8 (5.7)	24 (6.7)	0.678
Dyslipidaemia	22 (15.6)	19 (5.3)	< 0.001
Hypertension	22 (15.6)	38 (10.6)	0.120
Respiratory disease	8 (5.7)	17 (4.7)	0.665
Cardiovascular disease	7 (5.0)	5 (1.4)	0.019
Symptom category (at time of NIC admission)			
Asymptomatic	33 (23.4)	34 (9.5)	< 0.001
Pre-symptomatic	1 (0.7)	9 (2.5)	-
Symptomatic	89 (63.1)	279 (77.7)	-
Recovered	18 (12.8)	37 (10.3)	-
Disease severity			
Asymptomatic/mild	116 (82.3)	301 (83.8)	0.148
Moderate	17 (12.1)	34 (9.5)	-
Severe	3 (2.1)	19 (5.3)	-
Critical	5 (3.5)	5 (1.4)	-
Outcome			
Survival	138 (97.9)	354 (98.6)	0.556
Death	3 (2.1)	5 (1.4)	-

NIC: National Isolation Centre; SD: standard deviation.

Numbers in parentheses are percentages.

There was no difference in the mean duration of symptoms before admission between first and second wave cases (3.9 ± 3.6 vs 3.6 ± 2.6 days, respectively, *P* = 0.260). However, the mean time between specimen collection and admission to the NIC was shorter in the second wave than in the first (0.2 ± 1.0 vs 2.6 ± 2.0 days, respectively, *P* < 0.05).

**Fig. 1:**
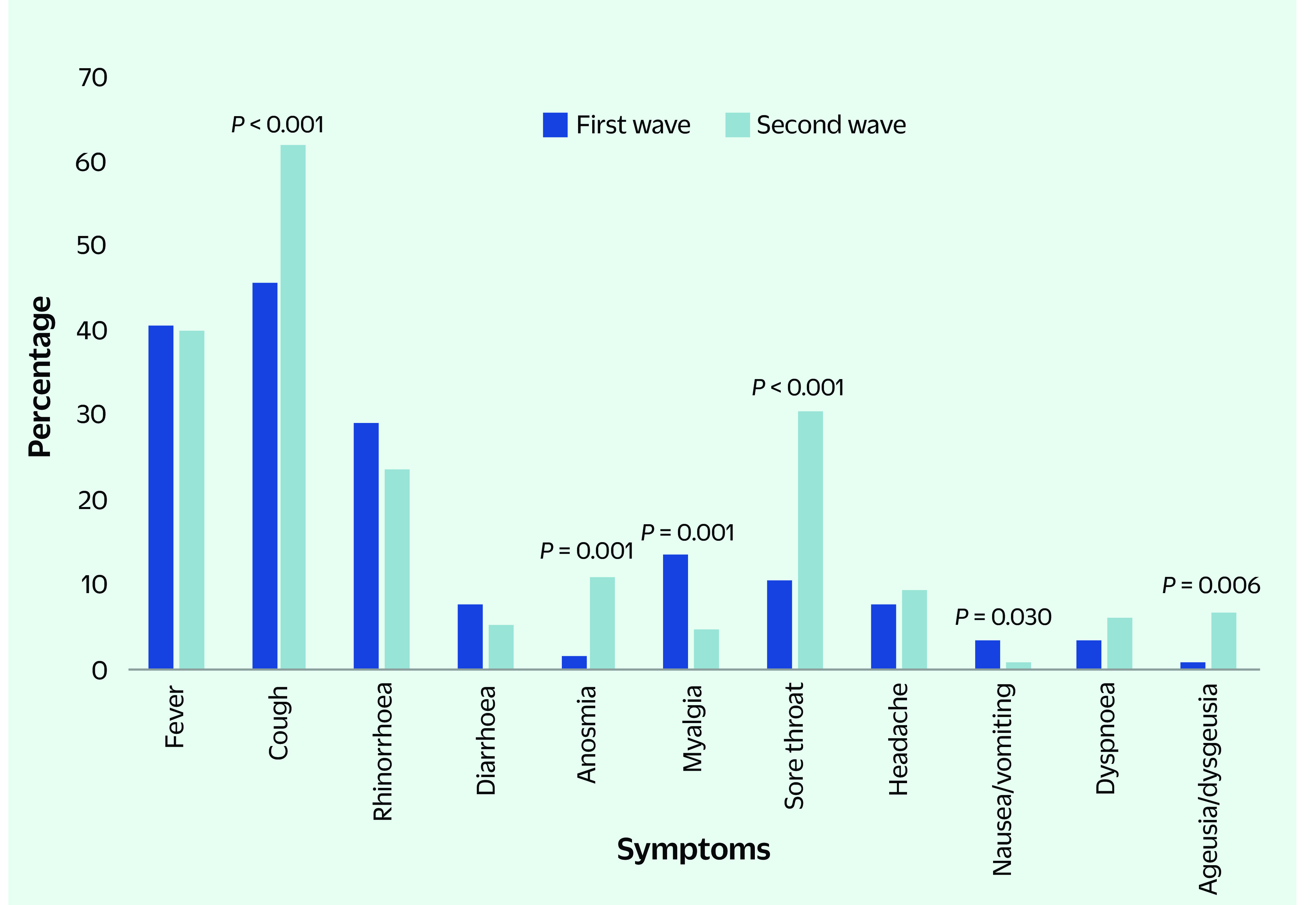
Comparison of symptoms reported at admission among COVID-19 cases in the first wave (n=141) and second wave (n=359), Brunei Darussalam, 2020–2021

A significantly higher proportion of second wave cases reported symptoms at admission compared with the first wave (77.7% vs 63.1%, respectively, *P* < 0.001). Relative to the first wave, patients were significantly more likely to report cough, anosmia, sore throat and ageusia/dysgeusia, but significantly less likely to report myalgia and nausea/vomiting (all *P* < 0.05) (**Fig. 1**). There was no difference in the number of symptoms reported between the first and the second waves (2.6 ± 1.4 vs 2.4 ± 1.2, respectively, *P* = 0.890).

[**insert** Figure 1]

The proportion of asymptomatic patients at admission was higher in the first wave (*P* < 0.001; **Table 1**). However, there was no significant difference between the two waves in terms of the distribution of cases by disease severity (*P* = 0.148; **Table 1**); in both waves, the majority of cases (> 80%) were categorized as either asymptomatic or mild.

During the first wave, none of the cases were vaccinated as vaccines against COVID-19 had not yet become available. Of the second wave cases, 13 (3.6%) were fully vaccinated (two doses), 41 (11.4%) were partially vaccinated (one dose) and 305 (85.0%) were unvaccinated. Of the unvaccinated cases, 117 (32.6%) were under the age of 18 years and not eligible for vaccination at the time. Most of the vaccinated cases had asymptomatic or mild disease, and there were more severe cases in the unvaccinated group (**Table 2**).

**Table 2:** COVID-19 cases in the second wave (admitted to the National Isolation Centre during 7–17 August 2021) by vaccination status and disease severity, Brunei Darussalam, 2021^a^

Vaccination status	All cases (*n* = 359)	Asymptomatic/mild (*n* = 301)	Moderate (*n* = 34)	Severe/critical (*n* = 24)
Vaccinated	13	12 (92.3)	1 (7.7)	0 (0)
Partially vaccinated	41	35 (85.4)	5 (12.2)	1 (2.4)
Unvaccinated	188	137 (72.9)	28 (14.9)	23 (12.2)
Ineligible	117	117 (100)	0 (0)	0 (0)

^a^ COVID-19 vaccination was not available during the first wave and therefore data are not presented for this group.

Numbers in parentheses are percentages.

## Discussion

Brunei Darussalam experienced a significant increase in the number of COVID-19 cases in its second wave of the pandemic in 2021, with nearly three times as many cases recorded just in the first 11 days than in the whole 2-month period of the first wave. This was not unexpected given that the Delta strain is more contagious than the original and has a higher R^0^. (*3*, *9*) In this respect, Brunei Darussalam’s experience is similar to other countries, with larger second waves widely reported. (*10*-*12*) For instance, a study in Thailand reported a sevenfold increase in case numbers between its first and second waves and another sevenfold increase between its second and third waves. (*10*)

As well as the substantial increase in the number of cases, this study has demonstrated distinct differences in the demographic profile of cases between the two waves. Cases in the second wave were significantly younger, with a lower proportion of cases occurring in people aged more than 50 years. One possible explanation for this shift to younger cases is the increase in case numbers among children following several outbreaks in schools in one of the country’s four districts at the start of the second wave. Other countries have also reported proportionally higher case numbers in the younger population in their second or subsequent waves, which some have attributed to acquired immunity as a result of SARS-CoV-2 infection, either diagnosed or undiagnosed during previous waves. (*13*, *14*) However, this is less likely to be true in the case of Brunei Darussalam as the first wave was quickly controlled and limited to just 141 confirmed cases when the last case of community spread was reported. While the possibility that undetected cases were circulating in the community cannot be ruled out, the numbers were likely to have been small.

Cases from the second wave were less likely to have comorbidities, which may simply be a reflection of the younger age of the second wave cohort. (*4*-*8*) Of the five specific comorbidities investigated, dyslipidaemia, cardiovascular disease, hypertension and respiratory disorders were more common among cases from the first wave, although the differences were only significant for dyslipidaemia and cardiovascular disease. Studies from Brazil, Japan, Spain and the United States of America have also reported similar differences between the first and second wave cases. (*4*-*8*) As well as being younger and having fewer comorbidities, second wave cases were less severe – exhibiting lower hospitalization rates, shorter lengths of stay, lower requirement for invasive mechanical ventilation and lower in-hospital mortality. (*5*)

In addition to demographic shifts, we also observed distinct differences in symptom burden. Compared with the first wave, the proportion of cases who reported symptoms at admission was significantly higher in the second wave, despite a shorter duration between symptom onset and NIC admission. However, there was no difference in the number of symptoms reported. Cough, anosmia, sore throat and ageusia/dysgeusia were significantly more common in second wave cases, whereas myalgia and nausea/vomiting were more likely to be reported by first wave cases, albeit in small numbers. It is unlikely that patients would have underreported symptoms such as anosmia and ageusia considering how uncommon and distressing these symptoms can be for patients.

Other studies conducted in the earlier part of the pandemic have reported variable but generally higher rates of symptoms than we found in our study. (*15*, *16*) A large meta-analysis which included data for over 60 000 patients reported that 87% of patients (95% confidence interval [CI]: 73–93, *P* < 0.001) had at least one COVID-19-related symptom. (*16*) Cough was reported by 68% of patients (95% CI: 56–74, *P* < 0.001); rates for other symptoms were as follows: fatigue, 39%; myalgia, 24%; dyspnoea, 24%; sore throat, 14%; headache, 14%; diarrhoea, 8%; rhinorrhoea, 7%; and nausea/vomiting, 6.5%. (*16*) However, studies included in this meta-analysis were limited to patients that presented for medical treatment or were hospitalized. In contrast, our study included patients from across the whole spectrum of COVID-19 disease severity, including mild and asymptomatic cases, and thus would be expected to yield a lower symptom rate. The proportion of patients who were asymptomatic accounted for almost a quarter of patients in the first wave and approximately 10% in the second wave. A further 12% had recovered by the time they were diagnosed and admitted for isolation, a proportion that remained largely unchanged between the first and second waves. Despite the differences in symptom burden between the first and second waves, the majority of cases were categorized as asymptomatic or mild, and fortunately, the proportion of critical cases remained low (< 5%).

The timing of the roll-out of the vaccination programme in Brunei Darussalam (*17*) meant that no first wave cases had been vaccinated, and by the end of the study period of the second wave, only 13 (3.6%) patients had been fully vaccinated (two doses). Despite the small sample size, our study provides some evidence that two doses of a COVID-19 vaccine conferred a benefit. Among the fully vaccinated group, over 90% of those who contracted COVID-19 had mild disease and there were no cases of severe/critical disease, whereas 23 of the 24 severe/critical cases in the second wave occurred in the unvaccinated group. Cases among children and adolescents, a group that was ineligible for vaccination during the period of our study, were all mild. This is consistent with other studies which also report that children and younger persons are more likely to have mild disease and are at low risk for mortality. (*18*, *19*)

To our knowledge this study is unique in that, due to our management policy which required all confirmed cases, including asymptomatic and mild cases, to be isolated (at least at the start of the second wave), we were able to compare the characteristics of two cohorts of COVID-19 cases, one from the first wave and the other from the second wave, both of which comprised cases across the spectrum of disease severity. Had we included patients admitted to the NIC after 18 August, we would have artificially shifted the profile of our second wave study cohort towards moderate, severe and critical cases. Since more severe cases are typically associated with older age and higher prevalence of comorbidities, as well as a greater frequency of co-infections, (*20*) this would have invalidated our comparisons. However, only including patients from the start of the second wave can itself be a limitation as any clinical and demographic shifts that occurred as the second wave progressed would not have been captured.

In conclusion, our study showed a distinct shift in the clinical and demographic characteristics of COVID-19 cases in terms of age, comorbidities and symptom burden between the first and second waves in Brunei Darussalam. This is similar to what has been reported in other countries. Knowledge of the changes in disease manifestations can help guide changes in management strategies, such as duration of isolation, testing strategies, and criteria for admission and treatment. Further studies will be required to assess if further shifts have occurred as the pandemic progressed.
